# Sedimentary structures of microbial carbonates in the fourth member of the Middle Triassic Leikoupo Formation, Western Sichuan Basin, China

**DOI:** 10.1038/s41598-023-28211-0

**Published:** 2023-02-09

**Authors:** Yuanchong Wang, Kaibo Shi, Bo Liu

**Affiliations:** 1 School of Electronic Information and Engineering, Yangtze Normal University, Chongqing, China; 2 Key Laboratory of Micro Nano Optoelectronic Devices and Intelligent Perception Systems, Yangtze Normal University, Chongqing, China; 3 Key Laboratory of Sedimentary Basin and Oil and Gas Resources, Ministry of Natural Resources, Chengdu, China; 4 School of Earth and Space Sciences, Peking University, Beijing, 100871 China

**Keywords:** Environmental sciences, Ocean sciences

## Abstract

In recent years, abundant natural gas has been found in microbial carbonates in the fourth member of the Leikoupo Formation in Western Sichuan Basin. In this study, from the observation of 626 microbial thin sections, four types of microbial carbonates are classified based on the differences of mesostructures. Among them, thrombolites and stromatolites are subdivided into eight types based on the differences of microstructures. Six types of microbial microstructure association (MSA) are identified, and are mainly developed in microbial mounds. The energy of sedimentary environment and hydrodynamic conditions of them from low to high is MSA-5, MSA-1, MSA-6, MSA-3, MSA-4 and MSA-2. Because of the arid climate in the Annie Period, a restricted platform are developed in the upper sub-member of the Leikoupo Formation in Western Sichuan Basin, and the sedimentary facies are lagoon (gypsiferous lagoon or salt lake in evaporate conditions), microbial mounds, shoals, inner platform shoals and open sea from the east to the west. Microbial microstructures not only affect the pore evolution of microbial carbonate reservoirs, but also affect the diagenesis of microbial carbonate reservoirs of the fourth member of the Leikoupo Formation.

## Introduction

Microbialites are formed by the trapping and binding of marl and/or detrital sediments of the benthic microbial communities, or by the inorganic or organic-induced mineralization associated with microbial activities^1–4^. Microbial carbonates, one common type of microbialites, were defined as the carbonates related to microbial growth, metabolism, properties of cell surface, and extracellular polymeric substances (EPS)^2,3^.

Nowadays, a typical microbial carbonate reservoir has been found in the fourth member of the Leikoupo Formation in the Western Sichuan Basin^5^. From 2008 to 2012, well CK-1 and XS-1 have produced commercial gas flow at the rate of 86.8 × 10^4^ m^3^/day and 68 × 10^4^ m^3^/day respectively. In 2014, well PZ-1 produced commercial gas flow at the rate of 121.05 × 10^4^ m^3^ /day. In next year, well YaS-1 and YS-1 produced commercial gas flow at the rate of 48.5 × 10^4^ m^3^ /day and 60.32 × 10^4^ m^3^/day respectively^6^. Although recent studies reached an agreement that the development of the microbial carbonate reservoir in the Leikoupo Formation was controlled by the sedimentary facies, there is also controversy over the sedimentary facies indentified by them^5,7–12^. Some researches indicated that the microbial carbonate reservoir in the Leikoupo Formation is developed in tidal flat^9,10^, while some preferred microbial reef^7,8,11^, and others were inclined to microbial mound^5^. Obviously, it is necessary for the exploration of microbial carbonate reservoir in the fourth member of the Leikoupo Formation in the Western Sichuan Basin to accurately restore the sedimentary environment of the microbial carbonates.

The formation of microbial carbonates is affected by sedimentary environment besides of microbial activities and metazoan activities^13^. Therefore, different types of microbial carbonates can indicate different sedimentary environments to a certain extent. For example, thrombolites, a common kind of microbial carbonates, are mainly developed in the subtidal zone with weak hydrodynamic force, which are usually observed as column, mound, lamination and thick shell^14^. Besides, the horizontally-laminated or wavy stromatolites are generally formed in tidal flat or lagoon with weak hydrodynamic force, while the large columnar and conical stromatolites are formed in the shallow subtidal zone with strong hydrodynamic force^13^. Obviously, the structures of microbial carbonates play an essential role in restoring the sedimentary environment.

Different types of microbial carbonates have different macrostructures, so many scholars classify microbial carbonates based on the differences in macrostructures. Aitken^15^ classified the macrostructure of microbial carbonates into thrombolites, stromatolites, cryptalgalaminites and oncolites. Kennard and James^16^ proposed the concept of mesoclots, and Burne and Moore^1^ classified the macrostructure of microbial carbonates into stromatolites, oncolites, thrombolites, cryptozoite and spherulite based on the classification of Kennard and James. Riding^2^ divides the macrostructures into stromatolites, thrombolites, dendrolites and leiolites. On the basis of the classification of Riding, Mei^17^ added oncolites and laminated rocks into the macroscopic structure. According to the biogenic classification of Embry and Kloven^18^, Han^13^ supplemented the epiphyton framestone and renalsis framestone into the macroscopic structure. Although microbial carbonates are roughly distinguished at macro-scale, they cannot reflect the structural differences at micro-scale. Therefore, Schmid^19^ proposed a classification based on the microfabric of the microbial carbonates, and three end members on the classification triangle chart are peloidal microstructure, laminated particle microstructure and dense microstructure. However, it cannot be reasonable and comprehensive if we classify the microbial carbonates based on the differences only in macro-scale or in micro-scale. Therefore, according to the differences of structure at multiple scales, the microbial carbonates should be classified at multiple scales. Shapiro^20^ proposed four scales of structures for the classification of microbial carbonates, including mega-structures (referring to the large-scale structures, such as biostrome), macrostructure (determining the morphology of microbial carbonate rocks, with a diameter ranging from centimeters to meters, for instance mounds, columns and domical hemispheroid), mesostructure (the macroscopic structures visible to the naked eye, e.g. lamellar, clotted and dendritic) and microstructure (microscopic features observed under a microscope or scanning electron microscope, for example peloids and filamentous microorganisms).

However, in recent studies, the classification for microbial carbonate rocks of the Leikoupo Formation is at meso-scale, which is divided into thrombolites, stromatolites and thrombolite stromatolites, without considering the micro-scale classification^7,8,11^. Therefore, in order to promote the exploration of the fourth member of the Leikoupo Formation in Western Sichuan Basin, in this study, microbial carbonates in the Leikoupo Formation will be classified at meso-scale and micro-scale, and the sedimentary environment will be discussed as well.

## Geologic setting

Sichuan Basin, which is located at the northwestern Yangtze Block, has experienced four tectonic evolution stages since Ediacaran: the rift stage (Ediacaran-Middle Permian), the subduction stage (Late Permian-Middle Triassic), the collision stage (Late Triassic) and the thrust-orogeny stage (Jurassic-Quaternary)^21–24^.

**Figure 1:**
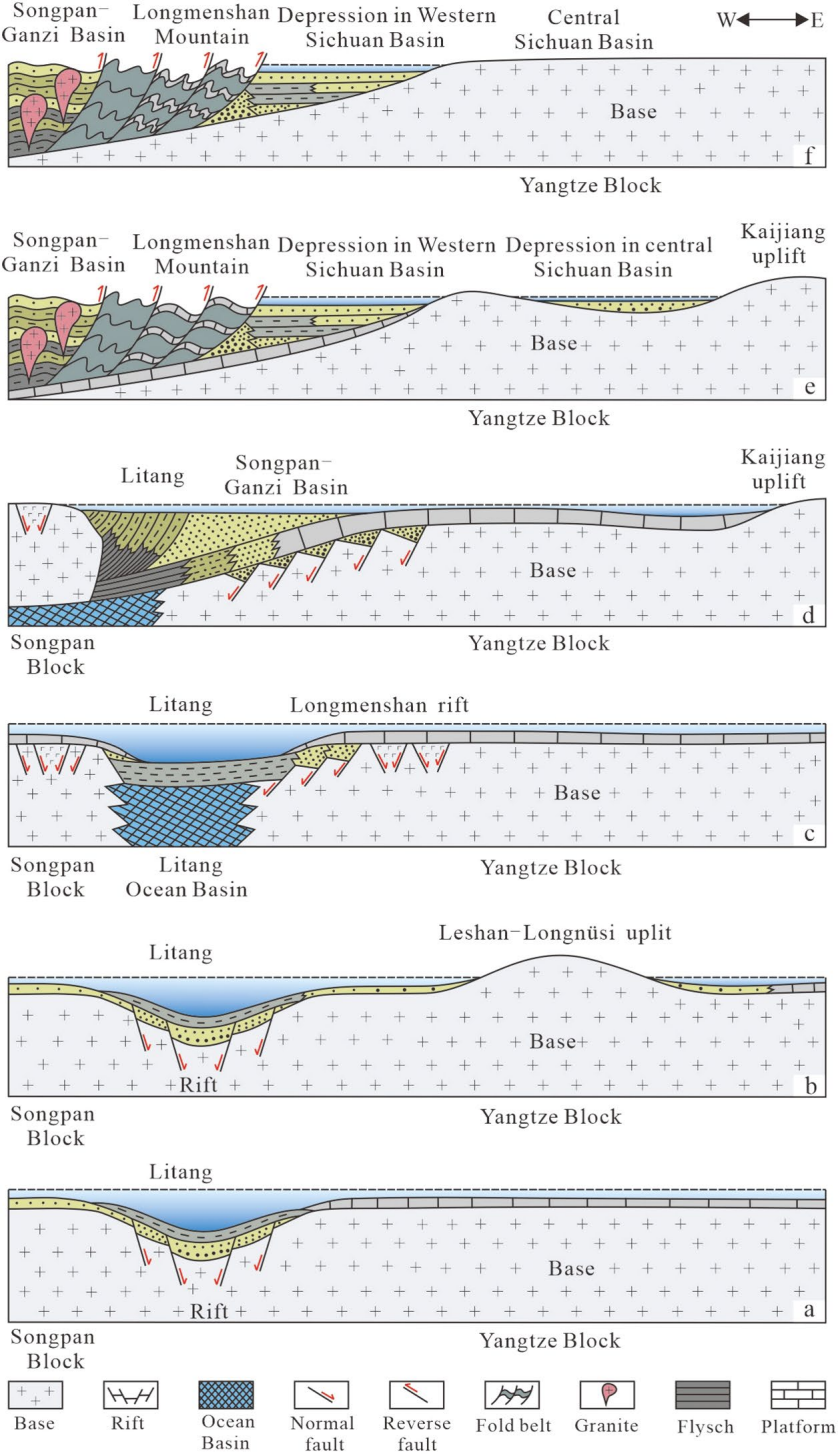
Tectonic evolution diagram of the western margin of the Yangtze Block (modified from He^21^ and Liu^22^. (**a**) The Sinian to the Middle Ordovician (635–458.4 Ma); (**b**) the Late Ordovician to the Silurian (458.4–416 Ma); (**c**) The Devonian to the Middle Permian (416–259.8 Ma); (**d**) The Late Permian to the Middle Triassic (259.8–237 Ma); (**e**) The Late Triassic (237–201.3 Ma); (**f**) The Jurassic to the Quaternary (201.3–0 Ma).

From the Sinian to the Middle Ordovician, the western margin of the Yangtze Block was adjacent to the Paleo-Tethys Ocean and was in an extensional tectonic regime for a long time. The Songpan Block in the west was attached to the Yangtze plate due to the Jinning movement, and an intraplate rift basin was developed between them. In the depression basin, stable neritic sediments developed inside the plate (Fig. 1a)^22,24^. From the Late Ordovician to Silurian, the extension of the western margin of the Yangtze Block continued to increase. While in the southeastern Yangtze Block, because of the collision between the Yangtze Block and the Cathaysian Block, a series of uplifts developed inside the Yangtze Block, such as the Leshan-Longnüsi uplift (Fig. 1b)^22,23^. At the beginning of the Devonian, the Yangtze Block entered the Paleo-Tethys stage. Before the Middle Permian, the extension between the western margin of the Yangtze Block and the Songpan Block continued to strengthen, forming the Jinshajiang-Lancangjiang ocean basin and the Litang ocean basin. The area of Longmen Mountain developed rifts (Fig. 1c)^21,22,24^.

From the Late Permian to the Middle Triassic, the tectonic regime changed from extension to compression. The two ocean basins Jinshajiang-Lancangjiang and Litang closed successively, and the Yangtze Block began to subduct under the Songpan Block in the west. The orogeny occurred (Fig. 1d), and the residual ophiolitic melange zone was developed, which led to the regression of the Paleo-Tethys Ocean along the NE-SW direction^22^. While in the Middle Triassic, the Paleo-Tethys Ocean did not completely withdraw from the Yangtze Block, and the carbonate platform was still developed.

In the Late Triassic, the Paleo-Tethys Ocean was completely closed, and the Songpan-Ganzi fold belt was formed on the western margin of the Yangtze Block, resulting in a strong extrusion thrust in direction of southeast. The early rift underwent tectonic inversion and normal faults were transformed into reverse fault, and the Songpan orogenic belt and the Longmen Mountain thrust nappe structure were formed (Fig. 1e). The Upper Triassic Xujiahe Formation with a thickness of more than 5 km was developed in clastic rocks^12,25,26^.

From the Jurassic to the Cretaceous, due to the continuous collision between the Songpan Block and the Yangtze Block, the Longmen Mountain imbricate thrust belt and foreland basin were developed in the western margin of the Yangtze Block, and entered into the uplift stage after the Paleogene (Fig. 1f)^21,22^.

**Figure 2:**
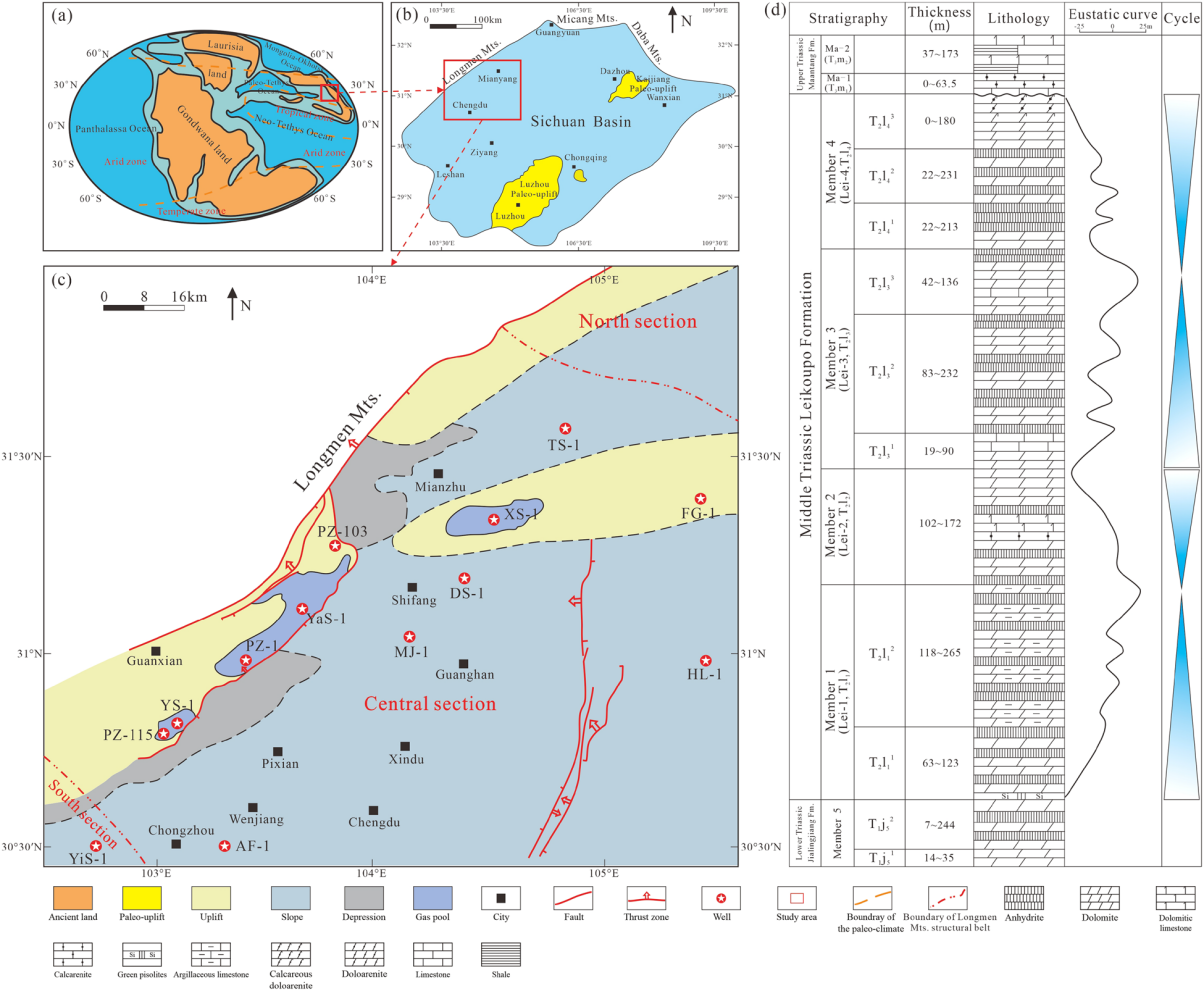
(**a**,**b**) Diagram of tectonic units of Sichuan Basin during the Middle Triassic Leikoupo deposition (**a** is modified from Wang^28^); (**c**) Diagram of present tectonic units and representative producing wells in Western Sichuan Basin (modified from Li^27^); (**d**) Sequence stratigraphy and lithology of the Leikoupo Formation in the Middle Triassic (modified from He^6^), T_2_l_1_^1^ = lower submember of the Lei-1; T_2_l_1_^2^ = upper submember of the Lei-1; T_2_l_2_ = Lei-2; T_2_l_3_^1^ = lower submember of the Lei-3; T_2_l_3_^2^ = middle submember of the Lei-3; T_2_l_3_^3^ = upper submember of the Lei-3; T_2_l_4_^1^ = lower submember of the Lei-4; T_2_l_4_^2^ = middle submember of the Lei-4; T_2_l_4_^3^ = upper submember of the Lei-4.

Obviously, the Middle Triassic was a special period of tectonic system transformation in the western margin of the Yangtze Block, and the sedimentary modern under tectonic control also underwent great changes. That is, before the end of the Middle Triassic, it was in the environment of passive continental margin developing carbonate platform, while from the late Triassic, clastic rocks in foreland basins were developed^6,21,23^. Due to the continuously thrust-orogeny, the present tectonic units of the Western Sichuan Basin are divided into ‘two uplifts (Longmen Mountain and Xinchang), two slopes (Guanghan-Zhongjiang and Wenxing-Mianyang) and two depressions (Yuantong-Ande and Mianzhu)’ (Fig. 2c)^27^.

The Middle Triassic in the Sichuan Basin is divided into Leikoupo Formation and Tianjingshan Formation from bottom to top. Influenced by the Indosinian movement, the Sichuan Basin was uplifted as a whole in the Late Triassic, resulting in the denudation of the Leikoupo Formation and the Tianjingshan Formation. Most areas in the eastern and southern Sichuan Basin show that the Leikoupo Formation is covered by the Upper Triassic strata unconformity. While in the Western Sichuan Basin, since the seawater had not completely withdrawn from the Yangtze Block, relatively complete Middle Triassic strata have developed, including Middle Triassic Leikoupo Formation, Tianjingshan Formation, Upper Triassic Ma'antang Formation, Xiaotangzi Formation (First Section of Xujiahe Formation) and Xujiahe Formation.

At present, the internal division of the Leikoupo Formation is relatively uniform. According to lithology and logging curve characteristics, it is divided into 4 members and 9 submembers, which contains Lei-1 (subdivided into T_2_l_1_^1^ and T_2_l_1_^2^), Lei-2, Lei-3 (subdivided into T_2_l_3_^1^, T_2_l_3_^2^ and T_2_l_3_^3^) and Lei-4 (subdivided into T_2_l_4_^1^, T_2_l_4_^2^ and T_2_l_4_^3^) (Fig. 2d)^6,12,28^.

## Samples and analytical method

A total of 626 core samples were obtained from 13 wells in study area (Fig. 2). 626 thin sections were stained by Alizarin Red S in one-third of the region to distinguish calcite and dolomite. Each thin section has a thickness of 0.03 mm impregnated with blue dye to recognize mega-pores.

## Results

### Microbial carbonates

Different types of microbial structures are quite different on the meso-scale. There aren’t obvious mega-scale and macro-scale microbial structures found in the field and core observations of the Lei-4. Thus, based on the classification of Mei^17^ and Flügel^14^, according to the different meso-scale structures, the microbial structures of the Lei-4 were classified, including thrombolites, stromatolites, dendrolites and oncolites. The two types of structures, thrombolites and stromatolite, are subdivided into eight categories because of the different microstructures (Table 1).

**Table 1:** Types of microbial carbonates in the fourth member of the Leikoupo Formation in Western Sichuan Basin.

Microbial meso-structure	Microbial micro-structure	Abbreviation	Thin section photography	Size of grains	Description	Water energy	Depositional environment
Thrombolites	Densed micrite thrombolites	DMT	Figure 3b	Micrite (clot) Silt (peloid and miliolinid )	Homogeneous marl	Low	Microbial mounds Lagoon
Thrombolites	Porphyritic micrite thrombolites	PMT	Figure 3c	Micrite (clot) Silt (peloid and miliolinid)	Porphyritic micrites that are darker than the marl	Low to medium	Microbial mounds Lagoon
Thrombolites	Peloidal-aggutinated thrombolites	PAT	Figure 3d	Micrite (clot) Silt (peloid and miliolinid) Fined (intraclasts)	A large number of peloids and few intraclasts in clots	Medium	Microbial mounds
Thrombolites	Foam laminated thrombolites	FLT	Figure 3e	Micrite (clot) Silt (peloid and miliolinid)	Stacking irregular bubble-like structures with dark surface	Medium to high	Microbial mounds
Stromatolites	Agglutinated thrombolitic stromatolites	ATS	Figure 3g	Micrite (lamina) Silt (peloid and miliolinid)	the transitional structure of thrombolites and stromatolites	Low to Medium	Microbial mounds
Stromatolites	Laminated fine-grained agglutinated stromatolites	LFAS	Figure 3h	Micrite (lamina) Silt (peloid and miliolinid)	Abundant peloids and few intraclasts in bright lamina	Low	Microbial mounds Lagoon
Stromatolites	Spongiostromate stromatolites	SS	Figure 3i	Micrite (lamina) Silt (peloid)	Same thickness of the dark laminae and the bright	Medium to high	Microbial mounds
Stromatolites	Skeletal stromatolites	SKS	Figure 3j	Micrite (lamina) Silt (peloid)	Both light and dark laminae are thin	High	Microbial mounds
Dendrolites	micritic coarse branching dendrolite	CBD	Figure 3k	Micrite (dendrolite) Silt (peloid)	Dendritic, dark branches and light gray dolomite in compartments	Low	Microbial mounds Lagoon
Oncolites	elliptic spherical concentric laminated oncolites	CLO	Figure 3l	Gravel (oncolites) Silt (peloid)	concentric laminations, while the core is composed of clots	Low	Microbial mounds Lagoon

#### Thrombolites

**Figure 3:**
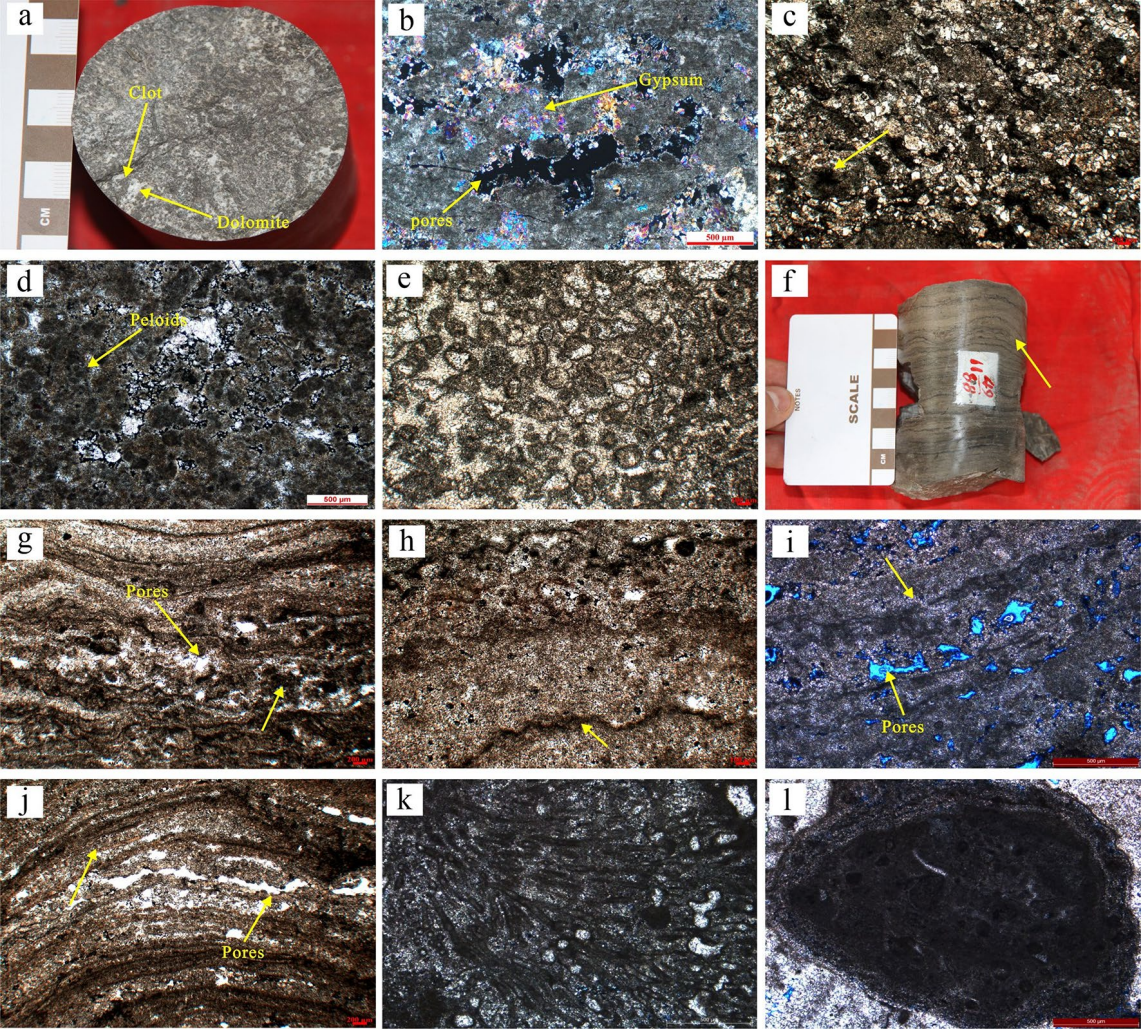
Macroscopic and microscopic characteristics of microbial carbonates in the Leikoupo Formation in Western Sichuan Basin. (**a**) Thrombolites, T_2_l_4_^3^, Well LS-1, 5985.20 m; (**b**) densed micrite thrombolites (DMT), T_2_l_4_^2^, Well TS-1,5843.80 m; (**c**) porphyritic micrite thrombolites (PMT)(yellow arrow), T_2_l_4_^3^, Well MJ-1, 6169.00 m; (**d**) Peloidal-aggutinated thrombolites (PAT), T_2_l_4_^3^, Well YS-1, 6235.90 m; (**e**) Foam laminated thrombolites (FLT), T_2_l_4_^3^, Well MJ-1, 6200.62 m; (**f**) Undulated lamina in stromatolites, T_2_l_4_^3^, Well LS-1, 5996.50 m; (**g**) Agglutinated thrombolitic stromatolites (ATS) (yellow arrow), T_2_l_4_^3^, Well YiS-1, 5886.80 m; (**h**) Laminated fine-grained agglutinated stromatolites (LFAS) (yellow arrow), T_2_l_4_^3^, Well YiS-1, 5891.28 m; (**i**) Spongiostromate stromatolites (SS) (yellow arrow), T_2_l_4_^3^, Well AF-1, 5706.70 m; (**j**) Skeletal stromatolites (SKS) (yellow arrow), T_2_l_4_^3^, Well YiS-1, 5886.80 m; (**k**) Micritic coarse branching dendrolite (CBD), T_2_l_4_^3^, Well YaS-1, 5776.73 m; (**l**) Elliptic spherical concentric laminated oncolite (CLO), T_2_l_4_^3^, Well AF-1, 5703.72 m.

At the meso-scale, thrombolites are characterized by dark gray, clotted, irregularly shaped (Fig. 3a). The sizes of the clots are ranged from 0.5 to 3 mm, and the space between the clots is commonly filled with dolomite or calcite. A large number of needle-like dissolved pores and dissolved pores can be found between the clots in some strata. At the micro-scale, four types of thrombolites can be identified as follows: (A), Densed micrite thrombolites (DMT), the interior of the clots is composed of homogeneous marl, and the intraclasts and peloids are almost invisible. In some sections, there is abundant gypsum developed between clots and in clots (Fig. 3b). It shows that the material bound by microorganisms is mainly marl, and the sedimentary environment is relatively quiet and the energy of the waterbody is low. (B), Porphyritic micrite thrombolites (PMT), beside of the marl inside the clot, there are porphyritic micrites that are darker than the marl (Fig. 3c), which may be related to the calcification of microorganisms. A small amount of intraclasts and peloids can be found. (C), Peloidal-aggutinated thrombolites (PAT), a large number of peloids and a small amount of intraclasts are developed in the clots. The sizes of the intraclasts range from 0.2 to 0.35 mm (Fig. 3d). Peloids in the thrombolites are rounded, micritic and uniformed, which accord with the characteristics of microbial peloids^14^. (D), Foam laminated thrombolites (FLT), the clots are mostly formed by stacking irregular bubble-like structures, and clean powder crystal dolomites are commonly filled in the bubbles. Some residual pores can be found in the bubbles, while the edge of the bubble is a dark gray micrite envelope (Fig. 3e). The formation process of foam laminated thrombolites is related to the microbial activities. Microorganisms release CO_2_ in the process of degrading organic matter. When CO_2_ escapes from the microbial mat, a bubble-like structure is formed, and calcite is precipitated on the surface of the bubble due to the calcification of microorganisms, forming the foam laminated structure^29^.

#### Stromatolites

At the meso-scale, stromatolites are characterized by the obvious laminar structures with undulating laminae and alternate development of bright and dark laminae. The thickness of a single alternation is about several millimeters (Fig. 3f). It indicates that stromatolites are usually developed in the turbulent waterbody^30^. When the energy of waterbody is high, bright laminae is developed because of the frequent circulation of fluid that leads to the sufficient cementation of clean calcite. While when it is low, dark laminae will show dominance since the circulation of fluid is limited, microbial structures can be well preserved. A large number of dissolved pores distributed along the lamina can be seen in the bright laminae in some layers. At the microscale, four types of stromatolites are mainly developed. (A), Agglutinated thrombolitic stromatolites (ATS), the transitional structure of thrombolites and stromatolites, are mainly composed of thrombolites with a certain orientation, and the laminae are not obvious. A few dissolved pores between the clots can be found, and the directionality is not strong (Fig. 3g). (B), Laminated fine-grained agglutinated stromatolites (LFAS), the dark laminae of which is irregular. The thickness of the dark laminae range from 0.05 to 0.1 mm, while the thickness of the bright laminae is more than 1 mm. A large number of peloids and a few intraclasts are bonded in it (Fig. 3h). (C), Spongiostromate stromatolites (SS), the thickness of the dark laminae and the bright laminae is much the same. The dark laminae are mostly composed of thrombolite, marl and peloids. The bright laminae are cemented with powder crystal dolomite and oriented dissolved pores can be seen (Fig. 3i). (D), Skeletal stromatolites (SKS), the thickness of both light and dark laminae are thin, indicating the quick changes of water energy. The oriented dissolved pores are developed between the laminae (Fig. 3j). The four types of stromatolites show that although the water energy is relatively high to the thrombolites, the frequency of water energy changes of these four types differs from each other. According to the microstructure, the SKS are the fastest, which are followed by SS, LFAS and ATS.

#### Dendrolites

At the meso-scale, dendrolites are commonly dendritic and shrublike. Under the microscope, the type of dendrolites is micritic coarse branching dendrolite (CBD). The width of a single branch ranges from 6 to 10 microns. Within the branches, it filled with micrite calcite or dolomite of light gray, while the surface of the branches is dark gray consisting of marl. Between the branches, there are elliptic or irregular structures like compartments filled with clean calcite or dolomite (Fig. 3k).

#### Oncolites

Oncolites are usually not obvious in core observation. While at microscopic scale, the oncolites in the Lei-4 are usually elliptic spherical concentric laminated oncolites (CLO), which are made up of core and coats. The size of the oncolites are greater than 2 mm. The coats are concentric laminations with irregular shape, which is probably affected by microbial activities, while the core is composed of clots and peloids (Fig. 3l).

### Other carbonates

Besides of microbial carbonates, grainstone, gypsum and crystal carbonates are developed in the Lei-4.

#### Grainstone

**Figure 4:**
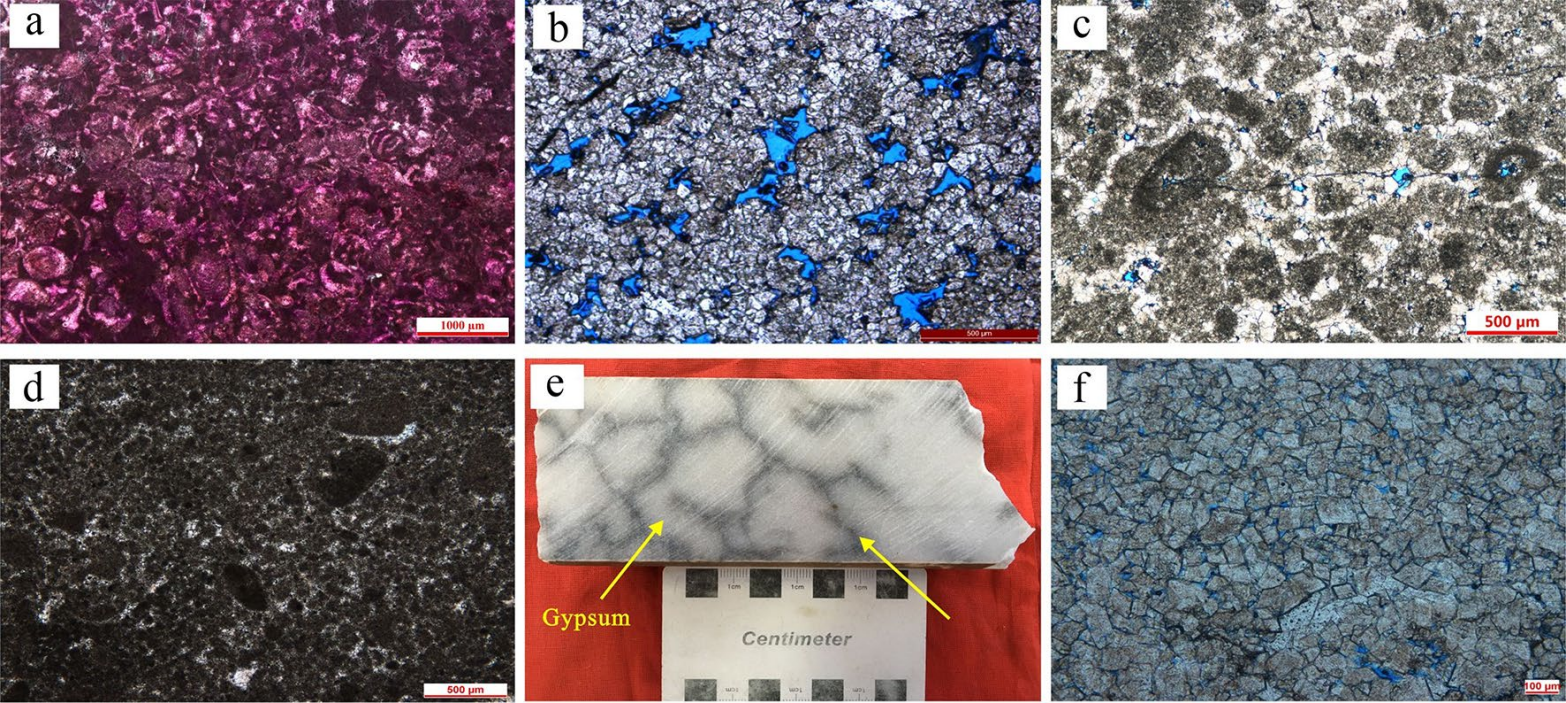
Macroscopic and microscopic characteristics of other carbonates in the Leikoupo Formation in Western Sichuan Basin. (**a**) Miliolinid packstone, T_2_l_4_^3^, Well YS-1,6127.39 m; (**b**) Intraclast grainstone, T_2_l_4_^3^, Well AF-1, 5695.88 m; (**c**) Intraclast grainstone, T_2_l_4_^3^, Well YaS-1, 5785.33 m; (**d**) peloid packstone to grainstone, T_2_l_4_^3^, Well YaS-1, 5785.90 m; (**e**) Gypsum with microbial related dark gray filamentous (yellow arrow), T_2_l_4_^3^, Well AF-1, 5622.50 m; (**f**) Crystal powder dolomite, T_2_l_4_^3^, Well YaS-1, 5789.92 m.

Grainstone in the Lei-4 are mainly developed in the T_2_l_4_^3^, including miliolinid packstone, intraclast grainstone and peloid packstone to grainstone. Microscopically, the grains are dominated by bioclasts, mainly benthic foraminifera and a small number of shell fragments. Few peloids can also be found. Inter-grains are filled with micritic calcite or dolomite (Fig. 4a). The miliolinid packstone is commonly developed in lagoons with low energy. Intraclast grainstone are dominated by intraclasts and a few peloids. The internal of the intraclasts is mainly made up of uniform marl and clots occasionally. A few elliptic peloids are developed between the intraclasts, which are composed of uniform marl. Sparry calcite and dolomite are cemented in the pores between the grains. A small number of intergranular pores and cracks were observed in some sections (Fig. 4b,c). Intraclast grainstone is commonly developed in shoal with high energy. Peloid packstone and grainstone is given priority to peloids and a few intraclasts are developed. Micritic calcite and dolomite are fully filled between the peloids (Fig. 4d), in the part of the sand dust as chondrules dolomite chip is a small amount of intergranular pore. The peloid packstone and grainstone are often associated with the intraclast grainstone and developed in lagoon and shoal^6^.

#### Gypsum

The climate of the Anisian stage was arid, which lead to the development of the gypsum in the T_2_l_4_^1^ and T_2_l_4_^2^^11^. The average thickness of gypsum in a single well is more than 200 m, while rare in the T_2_l_4_^3^. The gypsum is present as lamina, and plenty of dark gray filamentous reticular micritic dolomite is developed in it, which is related to microbial activities (Fig. 4e). Peloidal agglutinated thrombolite can also be found occasionally. Gypsum of the Lei-4 are commonly developed in the gypsiferous lagoon^6^.

#### Crystal carbonates

Crystal carbonates in the Lei-4 are divided into micritic calcite or dolomite and crystal powder dolomite. At micro scale, a few peloids are developed in micritic calcite or dolomite, and are mainly found in lagoon. While in crystal powder dolomite, the size of the dolomite ranges from 0.02 to 0.1 mm, and most of them are hypidiomorphic or xenomorphic with dirty surface (Fig. 4f). Residual densed micritic thrombolite structures can be found, which indicates that the crystal powder dolomite may be formed by the recrystallization of thrombolites. Crystal carbonates of the Lei-4 are mainly developed in lagoon and microbial mound.

### Microbial micro-structure association

Based on the vertical development of microbial carbonates in the Lei-4, six types of microbial micro-structure association (MSA) can be classified as follows.

**Figure 5:**
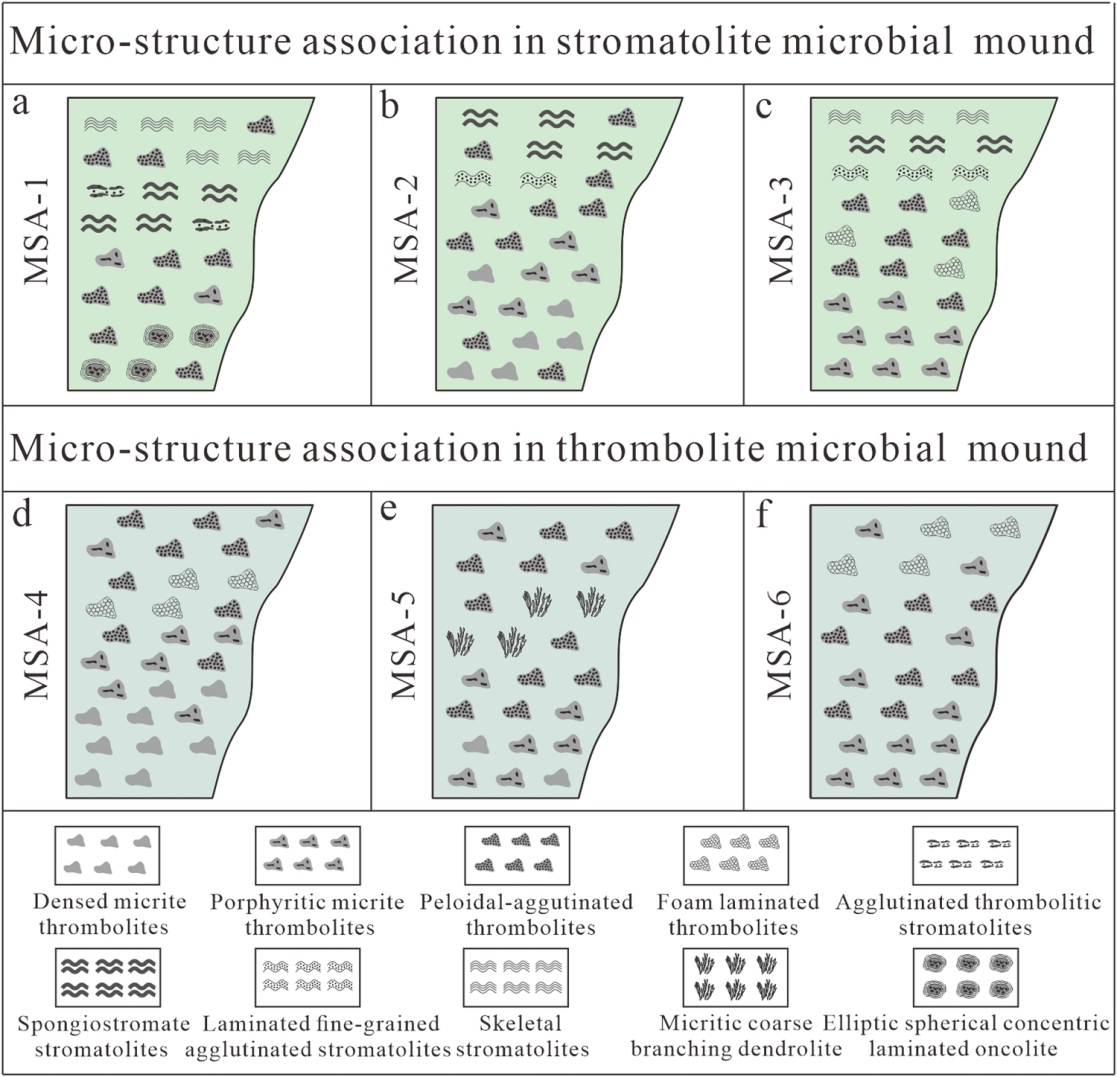
Characteristics of microbial micro-structure association in the Leikoupo Formation in Western Sichuan Basin.

**Figure 6:**
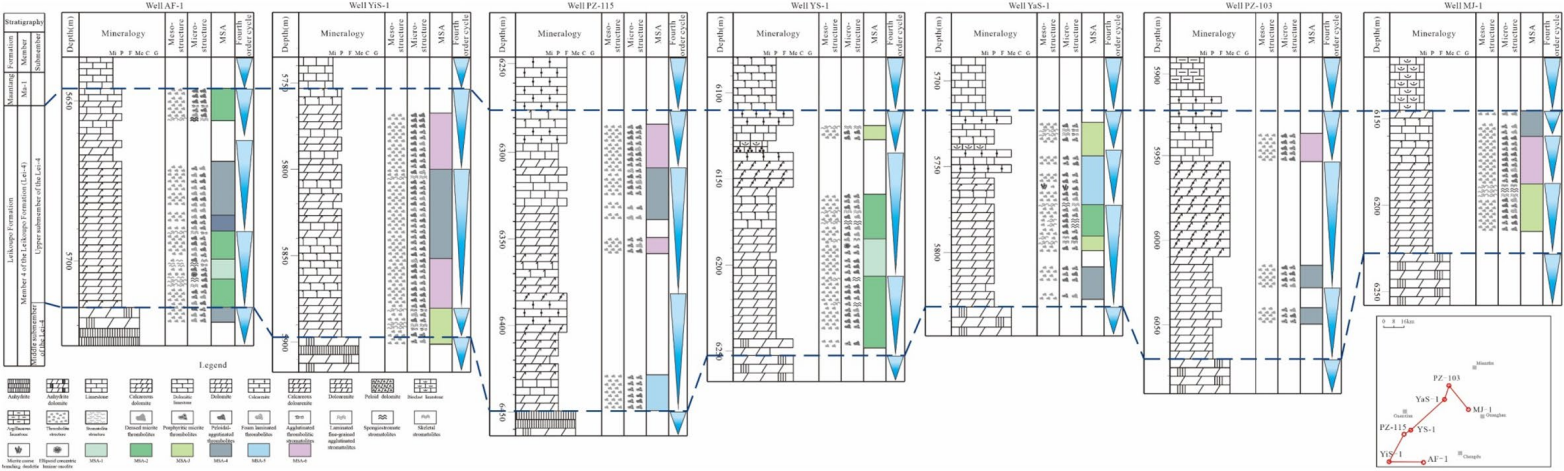
Vertical evolution and horizontal comparison of microbial micro-structure association in the Leikoupo Formation in Western Sichuan Basin.

MSA-1, mainly developed in stromatolite microbial mound, is composed of CLO, PAT, PMT, ATS, SS and SKS from bottom to top (Fig. 5). The MSA-1 in the Western Sichuan Basin is developed in the lower part of the T_2_l_4_^3^, and is mainly developed in well AF-1 and YS-1 (Fig. 6). Compared with other MSA, the MSA-1 is often developed in the lower or middle part of the fourth order cycle, which indicated that the energy of the environment of MSA-1 is low to medium.

MSA-2, also mainly developed in stromatolite microbial mound, is made up of DMT, PMT, PAT, LFAS and SS from bottom to top (Fig. 5). The MSA-2, mainly developed in the Well AF-1, YS-1 and YaS-1, can be found in the whole T_2_l_4_^3^ (Fig. 6). It is usually developed in the upper part of the fourth order cycle, showing the high energy of its sedimentary environment.

MSA-3, which can be found in stromatolite microbial mound, is composed of PMT, PAT, FLT, LFAS, SS and SKS from the bottom up (Fig. 5). The MSA-3 can be found in the whole T_2_l_4_^3^, and it is mainly developed in the well MJ-1, YaS-1 and YiS-1 (Fig. 6). The MSA-3 is mainly developed in the middle or upper part of the fourth order cycle, which indicated that the energy of the sedimentary environment of MSA-3 is medium to high.

MSA-4, mainly developed in thrombolite microbial mound, is made up of DMT, PMT, FLT and PAT from the bottom up (Fig. 5). MSA-4 is widely developed in the study area except well YS-1 and can be found in the whole T_2_l_4_^3^ (Fig. 6). MSA-4 is usually developed in the middle and upper part of the fourth order cycle, showing the medium to high energy of its sedimentary environment.

MSA-5, mainly found in thrombolite microbial mound, is comprised of DMT, PMT, CBD and PAT from bottom to top (Fig. 5). MSA-5 in the study area is found in the lower part of T_2_l_4_^3^ in well PZ-115 and middle part of T_2_l_4_^3^ in well YaS-1 (Fig. 6). MSA-5 is mainly developed in the lowest part of the fourth order cycle, which indicated that the energy of sedimentary environment of MSA-5 is the lowest compared to the others.

MSA-6, also mainly developed in thrombolite microbial mound, is made up of PMT, PAT and FLT from the bottom up (Fig. 5). MSA-6 is widely developed in the study area except well AF-1, YS-1 and YaS-1, and it can be found in the middle and upper part of the T_2_l_4_^3^ (Fig. 6). MSA-6 is often found in the lower and middle part of the fourth order cycle, showing the low to medium energy of its sedimentary environment.

It is obviously that the energy of sedimentary environment in the T_2_l_4_^3^ from low to high is MSA-5, MSA-1, MSA-6, MSA-3, MSA-4 and MSA-2.

## Discussion

### Sedimentary environments

#### Lagoon

The lithofacies of lagoon are dominated by micritic calcite or dolomite and crystal powder dolomite. The seawater circulation of lagoon is relatively blocked so that water energy is weak. The color of the carbonates in lagoon is dark gray and a few miliolinid foraminifera and fragments of shells can be found in it. Under evaporation conditions, salinization of seawater happened, the gypsiferous lagoon and salt lake are developed in the T_2_l_4_^1^and T_2_l_4_^2^, with a cumulative thickness of more than 300 m.

#### Microbial mound

A lot of anhydrite can be found in the microbial carbonates in the study area, not only in matrix, but also between frameworks, which shows that the microbial carbonates in the Lei-4 belongs to gypsiferous microbial carbonates (Fig. 3b). In modern examples, gypsiferous microbial carbonates are mainly developed in the shallow water of hot spring pools, evaporative lagoons and salt lakes, which are characterized by small mounds in morphology^31–33^. Therefore, considering of the evaporate climate in the Annie Period, microbial mound are developed in the Lei-4. The microbial mound in the study area are divided into thrombolite microbial mound and stromatolite microbial mound. Microbial mounds are mainly developed in the upper part of the fourth order cycle, and based on the analysis of MSA above, the seawater circulation and the hydrodynamic force of stromatolite microbial mound is relatively stronger than thrombolite microbial mound.

#### Shoal

The lithofacies of shoals are dominated by miliolinid packstone, intraclast grainstone and peloid packstone to grainstone. The seawater circulation of shoals is not blocked so that water energy is strong. The color of the carbonates in lagoon is light gray and a lot of peloids and microbial relative grains can be found in it. Shoals in the T_2_l_4_^3^ is usually associated by microbial mounds.

### Sedimentary model

In the Middle Triassic, the tectonic system of the Yangtze block changed from extension to compression, and the Longmenshan Mountain, Luzhou and Kaijiang underwater paleo-uplift were formed in the western and central Sichuan areas respectively. With the gradual enhancement of extrusion, the underwater paleo-uplift of Luzhou and Kaijiang in the central Sichuan Basin was gradually uplifted, and the paleo-uplift was exposed to the surface during the deposition of the Lei-4. Therefore, the restricted platform is developed in Western Sichuan Basin. Due to the arid paleoclimate, the seawater in restricted platform is subjected to strong evaporation sometimes, which led to the development of abundant gypsum and gypsiferous microbial carbonates.

Because of the evaporate environment, a large number of punctate or sheet-like microbial mounds with a small size are developed in the lagoons of the Lei-4, which are parallel to the coastline. In addition, the grainstone is often developed on top of the microbial carbonates, indicating that the shoals are developed on or around the mounds (Fig. 6). The petrologic characteristics of the Lei-4 in the field section (mainly in Hanwang section in Mianzhu and Gaodiancun section in Dayi) indicate that the sheet-like shoals are developed in the west of study area which is also parallel to the coastline.

**Figure 7:**
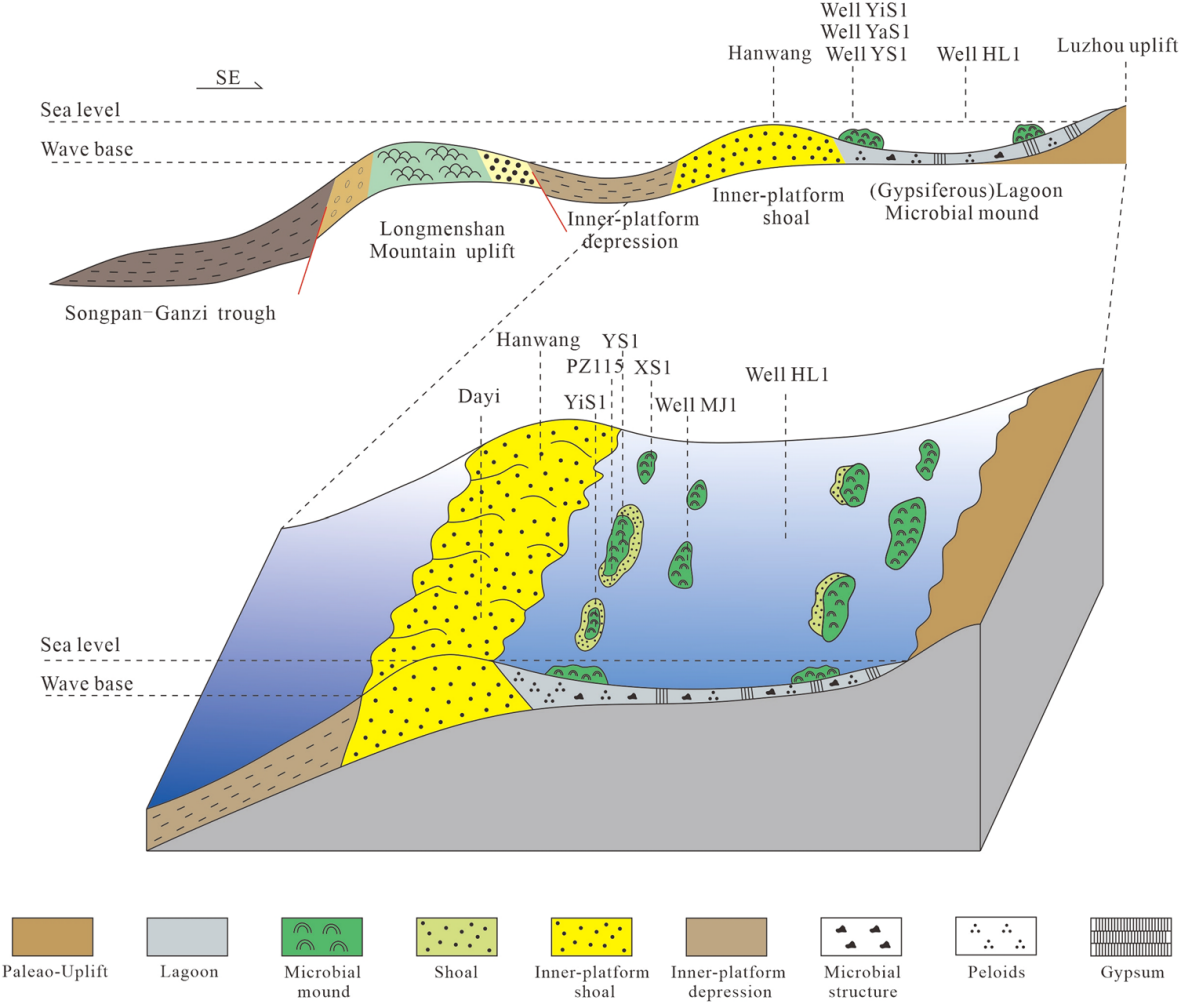
Sedimentary model of the Leikoupo Formation in Western Sichuan Basin.

Obviously, a restricted platform are developed in the study area, and the sedimentary faces are lagoon (gypsiferous lagoon or salt lake in evaporate conditions), microbial mounds, shoals, inner platform shoals and open sea from the east to the west (Fig. 7).

### Impact of micro-structure on microbial reservoirs of the Lei-4

Microbial micro-structure not only affects the pore evolution of microbial carbonate reservoirs, but also affects the diagenesis of microbial carbonate reservoirs of the Lei-4.

#### Impact of micro-structure on pore evolution

Pore evolution is very important for the formation of microbial carbonate reservoirs. Microbial carbonates are different from regular carbonates in that microbial activities have a great impact on sedimentary structure. Microbial carbonates are usually characterized by irregular sedimentary structures, diverse types and complex structures. The same macroscopic sedimentary structure, but the microscopic sedimentary structure is quite different. For example, thrombolites can be divided into densed micrite thrombolites(DMT), porphyritic micrite thrombolites (PMT), peloidal-aggutinated thrombolites (PAT) and foam laminated thrombolites (FLT). Stromatolites are divided into agglutinated thrombolitic stromatolites (ATS), Laminated fine-grained agglutinated stromatolites (LFAS), Spongiostromate stromatolites (SS) and Skeletal stromatolites (SKS). Different micro-structures not only determine the strength of compaction resistance, but also affect the circulation of diagenetic fluid, especially in the early diagenetic stage, thus affecting the strength of cementation and dissolution.

#### Impact of micro-structure on diagenesis

The diagenesis of microbial carbonates is closely related to their sedimentary structures. On the one hand, the sedimentary structure of microbial carbonates indicates different sedimentary environments, and the sedimentary environment and sedimentary geomorphology not only affect the types of microbial structures and the distribution scale of microbial carbonate rocks^30,34,35^, but also determine the diagenetic environment of microbial carbonates in the early diagenetic stage. It will affect the formation, transformation, scale and distribution of pores^36–39^. On the other hand, microbial carbonates are formed by microbial trapping and bonding marl and fine-grained debris^2,3^. In this process, microbial mineralization makes microbial carbonates obtain certain compaction resistance in the sedimentary or early diagenetic stage, which is closely related to their sedimentary structure. For example, the compaction resistance of thrombolites in the deposition period is stronger than stromatolites. In addition, different sedimentary structures have different fluid circulation capacity in the diagenetic stage, which affects the strength of cementation and dissolution. For example, in the thrombolite-related structure, the framework pores are usually developed. Due to the uncertainty and diversity of the shape of clots, the pores are irregular and often appear as isolated large pores, which indicates that the fluid circulation capacity is limited, and the multistage cementation of the original seawater occurred in the original pores. While in stromatolite-related structure, pores developed along the undulating lamina, indicating that the fluid circulation is strong, and dissolution and cementation are not affected.

Although the macro-structures are the same, the strength of diagenesis varies greatly in different micro-structures microbial carbonates. For example, among the four types of thrombolites, large isoloated pores developed between clots in DMT, and few micro-pores can be observed in clots, which shows the strong compaction resistance during early diagenetic period (Fig. 1a). While in PAT, the size of pores between clots is small, and clots are closely packed, which indicates that the compaction resistance is weak in the early diagenetic period (Fig. 1b). In addition, the diagenesis in different micro-structure stromatolites also varies. In LFAS, original pores are nearly full of multiple cementation (Fig. 1c). While a large number of dissolved pores are developed in the bright laminae of SKS (Fig. 1d).

Obviously, different micro-structures of microbial carbonates usually experience different strength of dissolution and cementation. However, in modern studies, scholars mainly discuss the causes of diagenetic evolution and reservoir formation through the macroscopic structures of microbial carbonates. In future researches, it is very important for the exploration of microbial carbonate reservoirs to carry out more and deeper studies on the differences in sedimentary environment, pore structure and diagenesis of different micro-structure microbial carbonates.

## Conclusions

Microbial carbonates of T_2_l_4_^3^ in Western Sichuan Basin are divided into thrombolites, stromatolites, dendrolites and oncolites. The two types of microbial carbonates, thrombolites and stromatolites, are subdivided into eight types based on the differences of microstructures.

Six types of microbial microstructure association (MSA) are classified, and are mainly developed in microbial mounds. The energy of sedimentary environment and hydrodynamic conditions of them from low to high is MSA-5, MSA-1, MSA-6, MSA-3, MSA-4 and MSA-2.

A restricted platform are developed in T_2_l_4_^3^ in Western Sichuan Basin, and the sedimentary faces are lagoon (gypsiferous lagoon or salt lake in evaporate conditions), microbial mounds, shoals, inner platform shoals and open sea from the east to the west.

Microbial microstructures not only affect the pore evolution of microbial carbonate reservoirs, but also affect the diagenesis of microbial carbonate reservoirs of the Lei-4.

## Data Availability

The datasets used and analyzed during the current study are available from the corresponding author on reasonable request.
